# Severe and persistent nerve palsy after ultrasound-guided continuous interscalene brachial plexus block in a teenager undergoing shoulder surgery: a case report

**DOI:** 10.1186/s40981-020-0315-1

**Published:** 2020-02-15

**Authors:** Hisashi Shio, Shinichi Sakura, Akihiro Motooka, Yasuo Sakai, Yoji Saito

**Affiliations:** 1 Department of Anesthesiology, Shimane University Faculty of Medicine, 89-1 Enya-Cho, Izumo City, Shimane 693-8501 Japan; 2 Surgical Operation Center, Shimane University Hospital, 89-1 Enya-Cho, Izumo City, Shimane 693-8501 Japan; 3 Department of Rehabilitation Medicine, Shimane University Faculty of Medicine, 89-1 Enya-Cho, Izumo City, Shimane 693-8501 Japan

**Keywords:** Neurological complication, Continuous interscalene brachial plexus block, Neurophysiological measurements, Magnetic resonance imaging, Rehabilitation

## Abstract

**Background:**

Although neurologic sequela is a recognized complication after interscalene brachial plexus block (ISB), there is a paucity of information on how severe and persistent neuropathy occurs and develops.

**Case presentation:**

A healthy high school soccer goalkeeper was scheduled for an arthroscopic Bankart repair. After continuous ISB for 2 days, sensation in the C5 and C6 areas and motor function did not return. With symptomatic drug treatment for neuropathic pain and rigorous rehabilitation, recovery of sensory loss and muscle weakness were gradually observed around 1 to 2 months after surgery. He returned to sport 1 year after surgery.

**Conclusion:**

This report is the first detailed description of a case who incurred severe and persistent nerve injury after continuous ISB yet recovered nearly fully to return to being an athlete. The present case should also underscore the importance of close observation after surgery in cases where a patient receives continuous ISB.

## Background

Although neurological complications are not uncommon sequela of interscalene brachial plexus block (ISB), severe and persistent neurologic injury (sensorimotor deficit lasting > 6 months) after continuous ISB is rare [1, 2]. Therefore, there is a paucity of information on how severe and persistent neuropathy occurs and develops.

We report a case of a teenager who incurred severe and persistent nerve injury after ultrasound-guided continuous ISB. Fortunately, after undergoing treatment for neuropathic pain and detailed neurophysiological measurements plus rigorous rehabilitation, this teen had nearly a full recovery of his athletic ability.

## Case presentation

The patient was a healthy male high school soccer goalkeeper who suffered from recurrent dislocation of the right shoulder, for which an arthroscopic Bankart repair was planned.

Prior to general anesthesia, continuous ISB using ultrasound-guided posterior approach [3] was performed. Under sterile conditions, a linear ultrasound transducer (HFL50x 15–6 MHz; FUJIFILM SonoSite Inc., Bothell, WA, USA) was used to identify the C5, C6, and C7 roots. After 1% mepivacaine was injected to anesthetize the skin, we used in-plane ultrasound guidance to insert a 10-cm, 18-gauge Contiplex Tuohy needle (B-Braun Ltd., Tokyo, Japan) and guided it through the middle scalene muscle. A bolus of 20 ml levobupivacaine (0.5%) was administered through the needle in the space between the middle scalene muscle and the brachial plexus sheath at a level between the C5 and C6 roots. Following injection, a catheter was introduced and advanced 3 cm in the space where the catheter tip position was confirmed with an injection of air under ultrasound visualization. The catheter was secured with a subcutaneous insertion length of 11 cm. During the procedure, ultrasound imaging showed no contact between the needle tip and the nerve roots, and no paresthesia was felt. Fifteen minutes later, a loss of cold sensation (using an ice cube) in the distribution of the C5 and C6 nerve roots was observed.

Postoperatively he received continuous infusion of levobupivacaine (0.125%) at a basal rate of 4 ml/h for 2 days, during which visual analog scale (VAS) pain scores were 0 mm both at rest and in motion at 24 and 48 h, postoperatively. After the continuous injection was stopped, sensation in the C5 and C6 areas and the motor function such as elbow flexion did not return, and numbness and pain in the C5 and C6 areas became apparent on the 6th day after surgery. Then, a strong, painful Tinel’s sign was elicited by percussion in the posterior triangle of the right neck with radiating pain in the C5 and C6 areas. Based on these findings, C5 and C6 brachial plexus palsies were clinically suspected. A hematoma around the brachial plexus was excluded by ultrasonography.

**Fig. 1:**
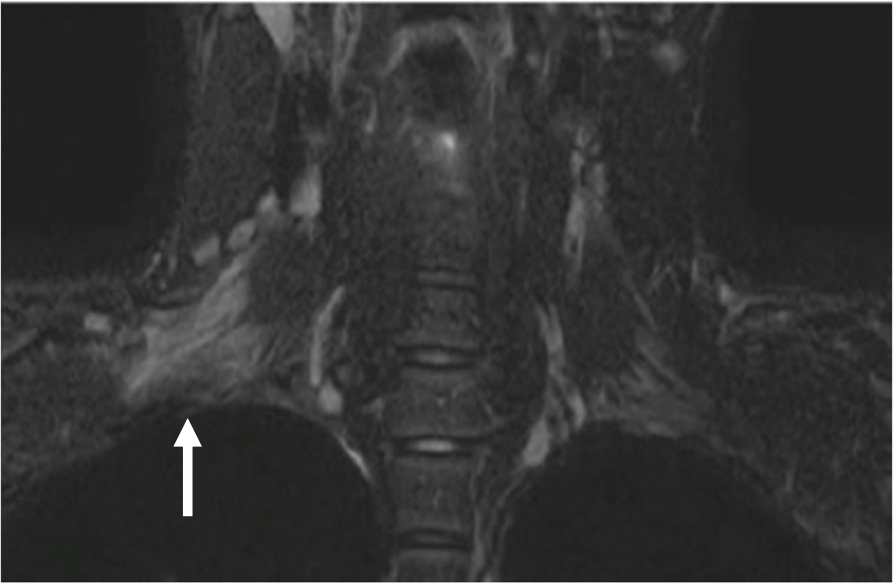
Coronal T2-weighted MRI of the neck at 1 week after surgery showing diffuse nerve enlargement and increased homogeneous signal intensity of the right brachial plexus (arrow). MRI indicates magnetic resonance imaging

**Fig. 2:**
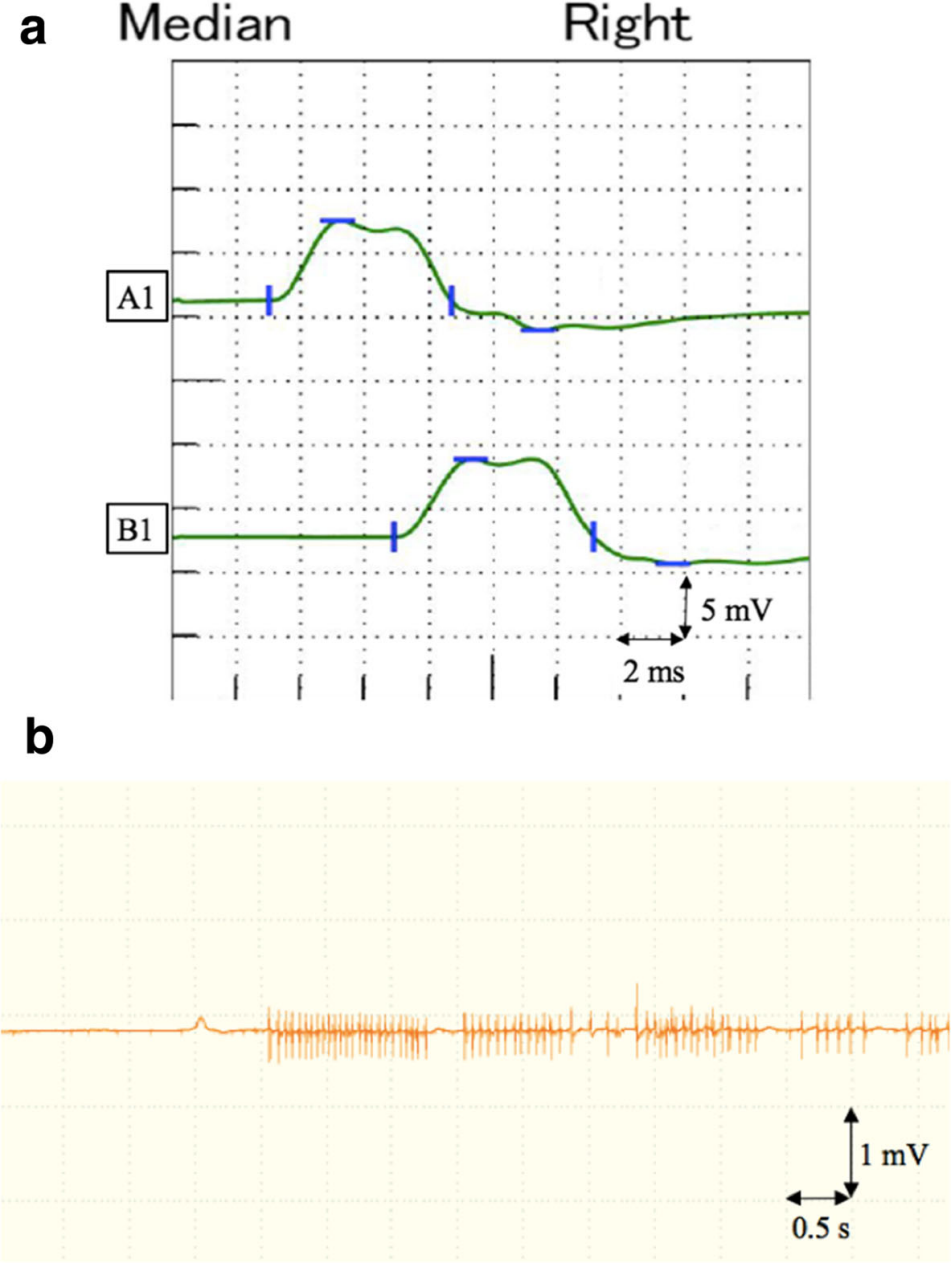
**a** Nerve conduction studies performed 2 weeks after surgery of the right median nerve with stimulations at the wrist (A1) and elbow (B1) showing reduced amplitude of compound muscle action potentials (8.5 mV and 8.2 mV, respectively). The latencies and nerve conduction velocities were normal. **b** Needle electromyography of the right biceps brachii muscle performed 3 weeks after surgery showing reduced interference pattern

**Fig. 3:**
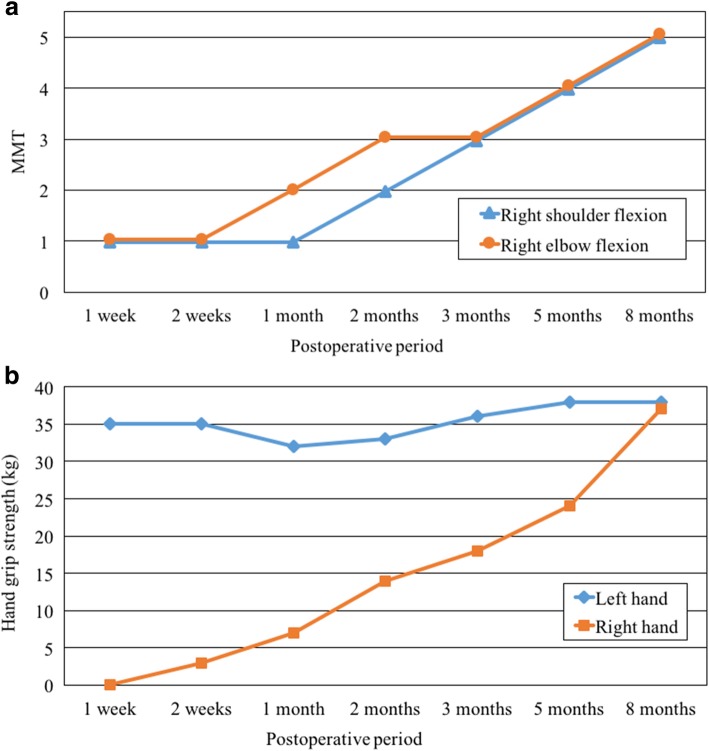
**a** The time course of postoperative MMTs of right shoulder and elbow flexion. MMT indicates muscle manual test. **b** The time course of postoperative hand grip strength

In addition to symptomatic treatment for numbness and pain with pregabalin and loxoprofen sodium hydrate, we conducted neurophysiological measurements, took a magnetic resonance imaging (MRI) examination, and started rehabilitation with passive range of motion exercises for the right shoulder and elbow joints as well as electrical muscle stimulation of paralyzed muscles. The MRI of the neck at 1 week after surgery showed diffuse nerve enlargement and homogeneous increased signal intensity of the right brachial plexus on T2-weighted images (Fig. 1). The results of the nerve conduction studies at 2 weeks after surgery showed reduced amplitude of compound muscle action potentials and sensory nerve action potentials of the right median nerve (Fig. 2a), both of which were suggestive of axonal degeneration, but did not reveal any abnormality of the right ulnar nerve. Needle electromyography at 3 weeks after surgery indicated that the injury site ranged from nerve roots distal to the origin of the dorsal scapular nerve to the nerve trunk and that the muscles such as the biceps brachii and the deltoid were not completely paralyzed based on the findings of motor unit potentials (MUPs), reduced interference pattern (Fig. 2b), and no sign of denervation. The numbness, pain, and Tinel’s sign improved about 2 weeks after surgery, but sensory loss and muscle weakness persisted. Signs of recovery were gradually observed around 1 to 2 months after surgery (Fig. 3a, b). Muscle strengthening exercises adjusted to the recovery of paralyzed muscles were continued. Both MRI and neurophysiological measurements performed 5 months after surgery showed dramatical improvement in the findings, and rehabilitation became more intense while aiming for a full return to being an athlete. At 8 months after surgery, the muscle strength and sensation improved to the same level as the non-paralyzed side. Finally, he returned to sport 1 year after the surgery.

## Discussion

Neuropathy after ISB occurs relatively frequently with the reported incidence around 2.84% [4]. However, the symptoms are mostly minor, and rarely is there a severe and persistent neurologic complication after continuous ISB. Previous studies [1, 2] have shown that the incidence of neurologic complications (sensorimotor deficit lasting > 6 months) ranges from 0 to 0.1%, but little information is available on how severe and persistent neuropathy occurs and develops. To the best of our knowledge, this report is the first detailed description of a case who incurred severe and persistent nerve injury after ultrasound-guided continuous ISB yet recovered nearly fully to return to being an athlete.

Perioperative nerve injury results from a multitude of clinical factors, which can be categorized in anesthetic, surgical, and patient factors [5]. The anesthetic factors can be further divided into mechanical factors associated with regional anesthesia or chemical factors related to the neurotoxicity of local anesthetics [6]. In the present case, the injury site estimated from neurophysiological measurements and MRI corresponded almost exactly with the distribution of local anesthetic by nerve block. In addition, our patient was a healthy teenager and the operation was performed uneventfully. Both an intraneural injection and a plexus compression due to hematoma were excluded by ultrasonography, but there is a possible involvement of other unknown factors. Therefore, we speculate that it is highly likely that local anesthetic continuously delivered to the nerve roots resulted in severe and persistent neuropathy together with other unknown factors.

In the present case, although local anesthetic was administered in a clinical dose, severe and persistent neuropathy still occurred. It is worthy of note that the patient felt no pain even when we moved his shoulder during 48 h. It is uncommon that pain intensity is so low after shoulder surgery even during continuous ISB. Our unpublished data show that VAS pain scores after arthroscopic shoulder surgery were 40 ± 31 (mean ± standard deviation) mm and 35 ± 21 mm at 24 and 48 h, respectively, on movement during continuous ISB using the same regimen as the present case. We should have noticed a possibility that something was going wrong when we checked the patient at 24 h. Studies have shown that all local anesthetics are potentially neurotoxic [7, 8] and that local anesthetic exhibits dose and time-dependent toxicity to nerves [7–9]. Therefore, it is possible that if we had discontinued the infusion earlier, i.e., at 24 h postoperatively, the damage might have been less serious.

Seddon classified nerve injuries into three major groups: neurapraxia, axonotmesis, and neurotmesis [10]. Sunderland proposed a more detailed classification [11], further dividing Seddon’s axonotmesis category into injuries with intact endoneurium, disrupted endoneurium but intact perineurium, and disrupted inner connective tissue layers with intact epineurium. These axonotmesis injuries of varying severity were designated types 2 to 4, from least to most severe. Sunderland referred to Seddon’s neurapraxia as a type 1 and neurotmesis as a type 5. It has been reported that when a large dose of lidocaine was administered continuously into the epidural space in rats, the specimen from the nerve roots showed edema, axonal degeneration, disintegrated myelin lamellae, and degenerated Schwann sheaths, with the continuity of the outer membrane of the fascicles being maintained [9]. These findings are equivalent to Sunderland type 3 injury in which recovery can occur over several months with conservative treatment or with surgical interventions to release entrapment sites [12]. From the clinical course and findings of neurophysiological assessments and MRI, we speculate that the nerve injury of this case is equivalent to a Sunderland type 3 injury. Despite previous reports [13, 14] showing poor recovery, in a case of severe and persistent neuropathy due to the toxicity of local anesthetic like this case, recovery can be expected with conservative treatment without surgical interventions. Therefore, it is important to continue treatment with hope of recovery without giving up.

During long-term management, maintaining motivation was crucial. Factors, such as having a clear goal of returning to play soccer, continuing to receive support from family and medical staff involved, confirming recovery process through detailed neurophysiological measurements, managing neuropathic pain, and rigorous rehabilitation, must have contributed to maintaining motivation and nearly a full recovery was observed 1 year after the incident.

## Conclusion

This report is the first detailed description of a case who incurred severe and persistent nerve injury after ultrasound-guided continuous ISB yet recovered nearly fully to return to being an athlete. The present case should also underscore the importance of close observation after surgery in cases where a patient receives continuous ISB.

## Data Availability

Data relevant to this case report are not available for public access because of patient privacy concerns but are available from the corresponding author on reasonable request.

## Abbreviations

ISB: Interscalene brachial plexus block
MMT: Muscle manual test
MRI: Magnetic resonance imaging
MUPs: Motor unit potentials
VAS: Visual analogue scale
